# Phenolic Compounds Known to Be Present in Lingonberry (*Vaccinium vitis-idaea* L.) Enhance Macrophage Polarization towards the Anti-Inflammatory M2 Phenotype

**DOI:** 10.3390/biomedicines10123045

**Published:** 2022-11-25

**Authors:** Riitta Ryyti, Mari Hämäläinen, Tiina Leppänen, Rainer Peltola, Eeva Moilanen

**Affiliations:** 1 The Immunopharmacology Research Group, Faculty of Medicine and Health Technology, Tampere University Hospital, Tampere University, 33014 Tampere, Finland; 2 Bioeconomy and Environment, Natural Resources Institute Finland, 96200 Rovaniemi, Finland

**Keywords:** macrophage polarization, low-grade inflammation, obesity, lingonberry, polyphenol, resveratrol, kaempferol, proanthocyanidin

## Abstract

Macrophages are pleiotropic immune cells whose phenotype can polarize towards the pro-inflammatory M1 or anti-inflammatory M2 direction as a response to environmental changes. In obesity, the number of macrophages in adipose tissue is enhanced, and they shift towards the M1 phenotype. Activated M1 macrophages secrete pro-inflammatory cytokines and adipokines involved in the development of systemic low-grade inflammation, complicating obesity. Polyphenols are widely found in the vegetable kingdom and have anti-inflammatory properties. We and others have recently found that lingonberry (*Vaccinium vitis-idaea* L.) supplementation is able to prevent the development of low-grade inflammation and its metabolic consequences in experimentally induced obesity. In the present study, we investigated the effects of twelve phenolic compounds known to be present in lingonberry (resveratrol, piceid, quercetin, kaempferol, proanthocyanidins, delphinidin, cyanidin, benzoic acid, cinnamic acid, coumaric acid, caffeic acid, and ferulic acid) on macrophage polarization, which is a meaningful mechanism determining the low-grade inflammation in obesity. Mouse J774 and human U937 macrophages and commercially available phenolic compounds were used in the studies. Three of the twelve compounds investigated showed an effect on macrophage polarization. Resveratrol, kaempferol, and proanthocyanidins enhanced anti-inflammatory M2-type activation, evidenced as increased expression of Arg-1 and MRC-1 in murine macrophages and CCL-17 and MRC-1 in human macrophages. Resveratrol and kaempferol also inhibited pro-inflammatory M1-type activation, shown as decreased expression of IL-6, NO, and MCP-1 in murine macrophages and TNF-α and IL-6 in human macrophages. In the further mechanistic studies, the effects of the three active compounds were investigated on two transcription factors important in M2 activation, namely on PPARγ and STAT6. Resveratrol and kaempferol were found to enhance PPARγ expression, while proanthocyanidins increased the phosphorylation of STAT6. The results suggest proanthocyanidins, resveratrol, and kaempferol as active constituents that may be responsible for the positive anti-inflammatory effects of lingonberry supplementation in obesity models. These data also extend the previous knowledge on the anti-inflammatory effects of lingonberry and encourage further studies to support the use of lingonberry and lingonberry-based products as a part of a healthy diet.

## 1. Introduction

Macrophages are pleiotropic immune cells, which have a central role in inflammation and host defense, as well as in the clearance of dead cells, wound healing, and repair [1]. Macrophages secrete cytokines and various other factors [2,3], and depending on the environment, they can polarize from a dormant state to a “classically activated” proinflammatory (M1) or to an “alternatively activated” anti-inflammatory (M2) phenotype [3,4,5,6]. M2-type macrophages are involved in the maintenance of tissue homeostasis, while M1-type macrophages possess various proinflammatory effector functions [7].

Macrophages are also present in adipose tissue. In lean adipose tissue, macrophages are mostly in the M2 state and release anti-inflammatory mediators [2,5,8,9,10]. In obesity, adipocytes grow, and they suffer from hypoxia and stress [11]. The environment supports macrophage infiltration into adipose tissue and their polarization towards the pro-inflammatory M1 phenotype [9,12]. The role of M1-type macrophages in adipose tissue is to clear dead adipocytes and other cell debris, secrete inflammatory cytokines, exocytose excess lipid, and contribute to adipose tissue remodeling [2,8,9,13,14]. The M1 polarization of adipose tissue macrophages is an essential factor promoting pro-inflammatory cytokine production, insulin resistance, and systemic inflammation associated with obesity [11,15,16]. Typical intracellular signaling pathways mediating macrophage polarization towards the M1 phenotype are the nuclear factor kappa B (NF-κB) and C-Jun N-terminal kinase (JNK) pathways [5,17]. Instead, signal transducer and activator of transcription 6 (STAT6) and peroxisome proliferator-activated receptor gamma (PPARγ) are significant transcription factors supporting polarization towards the M2 phenotype [17,18,19].

Dietary choices are important to find non-medical means to prevent the obesity-induced systemic low-grade inflammation and its metabolic comorbidities [9,20,21]. Polyphenols, especially anthocyanins and proanthocyanidins in berries, have been shown to attenuate M1-type macrophage activation and suppress inflammation [22,23,24,25,26]. In previous studies [27,28], we found that lingonberry (*Vaccinium vitis-idaea* L.) supplementation decreased low-grade inflammation in a high-fat diet-induced obesity in a mouse model. Lingonberry is known to contain polyphenolic compounds such as anthocyanidins, quercetin, kaempferol, resveratrol, proanthocyanidins, and phenolic acids [29,30,31,32], and the potential health benefits of lingonberry have been connected to its diverse polyphenol composition [22]. The aim of the present study was to investigate the effects of phenolic compounds previously identified to be present in lingonberry on macrophage M1/M2 polarization, which is an important mechanism regulating obesity-related low-grade inflammation.

## 2. Materials and Methods

### 2.1. Chemicals

Quercetin, kaempferol, benzoic acid, proanthocyanidins, delphinidin chloride, and cyanidin chloride were purchased from Carbosynth (Berkshire, UK) and resveratrol from Tocris Biotechne (Abingdon, UK). All other reagents were from Sigma-Aldrich (St. Louis, MO, USA), unless otherwise stated.

### 2.2. Cell Culture

Murine J774 macrophages (American Type Culture Collection (ATCC), Manassas, VA, USA) were cultured at 37 °C in a 5% CO_2_ atmosphere in Dulbecco’s modified Eagle’s medium (DMEM) with Ultraglutamine 1 (Sigma-Aldrich) adjusted to contain 10% heat-inactivated fetal bovine serum (FBS; R&D Systems Europe Ltd., Abingdon, UK), 100 U/mL penicillin, 100 μg/mL streptomycin, and 250 ng/mL amphotericin (all from Gibco, Thermo Fisher Scientific, Waltham, MA, USA). Cells were cultured in 24-well plates, and confluent cultures were exposed to fresh culture medium containing the compounds of interest. The cells were activated towards the M1 or M2 phenotype by adding bacterial lipopolysaccharide (LPS, 10 ng/mL) or interleukin 4 (IL-4, 1 ng/mL), respectively.

Human U-937 monocytes (ATCC) were cultured at 37 °C in a 5% CO_2_ atmosphere in RPMI 1640 medium adjusted to contain 2 mM L-glutamine, 20 mM HEPES, 4.5 g/L glucose (all obtained from Sigma-Aldrich), 1.5 g/L sodium bicarbonate, 1 mM sodium pyruvate, 100 U/mL penicillin, 100 μg/mL streptomycin, 250 ng/mL amphotericin (all obtained from Gibco), and 10% heat-inactivated FBS (BioWest, Nuaillé, France). Cells were seeded into 24-well plates and treated for 72 h with phorbol myristate acetate (PMA, 10 nM) to differentiate them into macrophages. Thereafter, the cells were serum-starved with 1% FBS for 16 h before experiments were commenced by adding the compounds of interest to fresh culture medium (with 1% FBS and supplements as above). The cells were activated towards the M1 or M2 phenotype by adding LPS (10 ng/mL) or IL-4 (10 ng/mL), respectively.

The toxicity of the investigated compounds in the current experimental conditions was assessed by a modified XTT test, which measures the mitochondrial dehydrogenase activity (Cell Proliferation Kit II, Roche Diagnostics, Mannheim, Germany). The assay is based on the cleavage of the tetrazolium salt XTT to form a formazan dye, which only happens in viable cells. Cells were grown for 72 h in a 96-well plate, and thereafter treated with the compounds of interest in the presence or absence of IL-4 or LPS for 21 h. The XTT reagent was added to the culture, and the incubation was continued for another 3 h. Cell viability was assessed by measuring absorbance at 490 nm (to detect the formazan dye), and 80% viability compared to control was set as a limit for cytotoxicity. Triton-X (0.1%) was used as a positive control for cytotoxicity. None of the compounds used in this study were found to be toxic.

### 2.3. RNA Extraction and Reverse Transcription Polymerase Chain Reaction 

Cells were incubated in the presence or absence of IL-4 (R&D Systems Europe Ltd., Abingdon, U.K.) and treated with the compounds of interest. Thereafter, cells were lysed at the indicated time points, and total RNA was extracted with the GenElute™ Mammalian Total RNA Miniprep Kit (Sigma-Aldrich), transcribed to cDNA using TaqMan Reverse Transcription reagents (Applied Biosystems, Foster City, CA, USA), and subjected to quantitative PCR with TaqMan Universal Master Mix (Thermo Fisher Scientific) and an ABI 7500 Real-Time PCR system (Applied Biosystems). The following TaqMan Gene Expression Assays (Thermo Fisher Scientific) were used: mouse MRC1 (Mm00485148_m1) and PPARγ (Mm01184322_m1) and human CCL17 (Hs00171074_m1) and MRC1 (Hs00267207_m1). The primers and probe for mouse Arg-1 were TCCAAGCCAAAGTCCTTAGAGATTAT (forward), CGTCAACTCTGTTTCTTTAAGTTTTTCC (reverse) and CGCCTTTCTCAAAAGGACAGCCTCGA (probe); for mouse GAPDH GCATGGCCTTCCGTGTTC (forward), GATGTCATCATACTTGGCAGGTTT (reverse) and TCGTGGATCTGACGTGCCGCC (probe); for human GAPDH AAGGTCGGAGTCAACGGATTT (forward), GCAACAATATCCACTTTACCAGAGTTAA (reverse) and CGCCTGGTCACCAGGGCTGC (probe). Results for mouse Arg-1 and both mouse and human GAPDH were calculated using the standard curve method, and for all others, the delta-delta CT method was used. All mRNA levels were normalized against GAPDH.

### 2.4. Protein Extraction and Western Blotting

Cells were incubated in the presence or absence of IL-4 and treated with the compounds of interest for the indicated time. Thereafter, cells were solubilized in cold lysis buffer (10 mM Tris-base, pH 7.4, 5 mM EDTA, 50 mM NaCl, 1% Triton X with 1 mM AEBSF, 0.8 µM aprotinin, 50 µM bestatin, 15 µM E-64, 5 µM EDTA, 20 µM leupeptin, 10 µM pepstatin A (Halt™ Protease Inhibitor Cocktail, Thermo Fisher Scientific), 1 mM sodiumorthovanadate, 2 mM sodiumpyrophospate, and 10 µM n-octyl-β-D-glucopyranoside) and incubated on ice for 20 min. Lysates were centrifuged (13,400× *g*, 4 °C, 10 min), and supernatants were collected and stored at −20 °C. Protein content in the samples was determined with the Coomassie blue method [33].

Phospho-STAT6, STAT6, and actin protein levels were detected using SDS-polyacrylamide gel electrophoresis and Western blot analysis as previously described [34] with antibodies #56554 for phospho-STAT6 and #9362 for STAT6 from Cell Signaling Technology (Beverly, MA, USA) and sc-1616R for actin from Santa Cruz Biotechnology (Santa Cruz, CA, USA). 

### 2.5. ELISA and NO Measurements in Cell Culture Media

The concentrations of mouse MCP-1 and IL-6 and human TNF-α and IL-6 in cell culture media were measured with the enzyme-linked immunosorbent assay (ELISA) using reagents from Thermo Fisher Scientific (human IL-6) and R&D Systems (other cytokines). Nitric oxide (NO) production was assessed by measuring nitrite, a stable metabolite of NO, in the cell culture media by the Griess reaction [35].

### 2.6. Statistical Analysis

The results are expressed as the mean ± the standard error of the mean (SEM). Unpaired *t*-test or one-way analysis of variance (ANOVA) with Dunnett’s post-test was used in the statistical analysis. Differences were considered significant at *p* < 0.05. Data were analyzed using the Prism computerized package (Graph Pad Prism Software, version 8, San Diego, CA, USA).

## 3. Results

### 3.1. Resveratrol, Kaempferol, and Proanthocyanidins Enhance M2-Type Activation in J774 Macrophages

**Figure 1:**
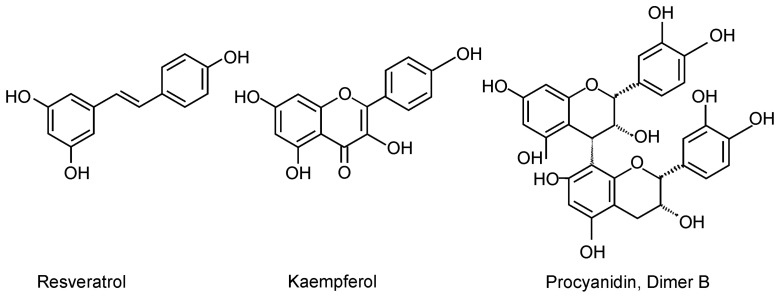
Chemical structures of resveratrol, kaempferol, and proanthocyanidins (procyanidin dimer B as an example).

**Figure 2:**
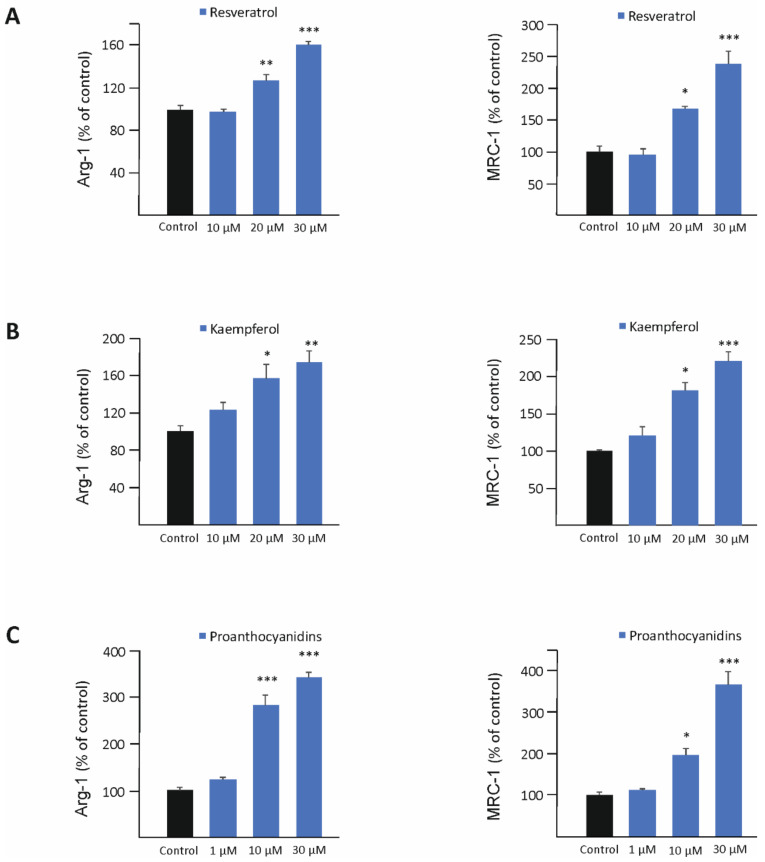
Resveratrol (**A**), kaempferol (**B**), and proanthocyanidins (**C**) enhanced the expression of arginase-1 (Arg-1) and mannose receptor C-type 1 (MRC-1) in murine J774 macrophages in a dose-dependent manner. Cells were cultured with resveratrol, kaempferol, or proanthocyanidins for 24 h. Expressions of Arg-1 and MRC-1 were measured by RT-PCR and normalized against GAPDH mRNA. Arg-1/MRC-1 expression in untreated cells was set as 100% (marked as black bar), and the other values are presented in proportion to that value. Results are given as the mean + SEM, *n* = 4. One-way ANOVA with Dunnett’s post-test was used in the statistical analysis. Mean values significantly different from the control are marked with * *p* < 0.05, ** *p* < 0.01, and *** *p* < 0.001.

The effects of twelve phenolic compounds known to be present in lingonberry were investigated on M2-type macrophage activation in murine J774 cells, including stilbenoids resveratrol and piceid, flavonols quercetin and kaempferol, proanthocyanidins, anthocyanidins delphinidin and cyanidin, and phenolic acids benzoic acid, cinnamic acid, coumaric acid, caffeic acid, and ferulic acid (Table 1). Cells were treated with the compounds of interest (30 µM), and their effect on the expression of two prototypic M2 markers, arginase-1 (Arg-1) and mannose receptor C-type 1 (MRC-1), was measured. Out of the twelve compounds studied, resveratrol, kaempferol, and proanthocyanidins significantly enhanced the expression of Arg-1 and MRC-1 (Table 1, Figure 1). In the further studies, J774 cells were treated with increasing concentrations of resveratrol, kaempferol, and proanthocyanidins, and all three compounds were found to enhance the expression of Arg-1 and MRC-1 in a dose-dependent manner (Figure 2).

The effects of resveratrol, kaempferol, and proanthocyanidins were studied also in IL-4-activated J774 macrophages. IL-4 (1 ng/mL) treatment enhanced Arg-1 expression by 23-fold and MRC-1 expression by 7-fold compared to untreated cells. All three compounds of interest at a 30 µM concentration significantly enhanced the expression of Arg-1 in IL-4-stimulated cells. Resveratrol and proanthocyanidins were also found to significantly elevate the expression of MRC-1, while the effect of kaempferol failed to reach statistical significance (Table 2).

### 3.2. Resveratrol and Kaempferol Increase the Expression of PPARγ, while Proanthocyanidins Enhance STAT6 Phosphorylation

**Figure 3:**
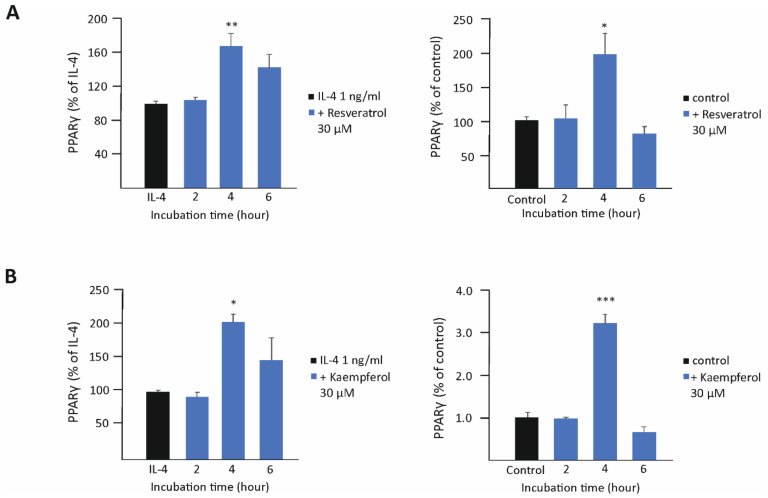
Resveratrol (**A**) and kaempferol (**B**) enhanced the expression of peroxisome-proliferator-activated receptor gamma (PPARγ) in murine J774 macrophages cultured with interleukin 4 (IL-4, (**left**)) or without IL-4 (**right**). Expression of PPARγ mRNA was measured by RT-PCR and normalized against GAPDH mRNA. PPARγ expression in the IL-4-treated cells ((**left**) column) or in the unstimulated cells ((**right**) column) was set as 100% (marked as black bar), and the other values are presented in proportion to that value. Results are given as the mean + SEM, *n* = 3–4. One-way ANOVA with Dunnett’s post-test was used in the statistical analysis. Mean values significantly different from the control are marked with * *p* < 0.05, ** *p* < 0.01, and *** *p* < 0.001.

**Figure 4:**
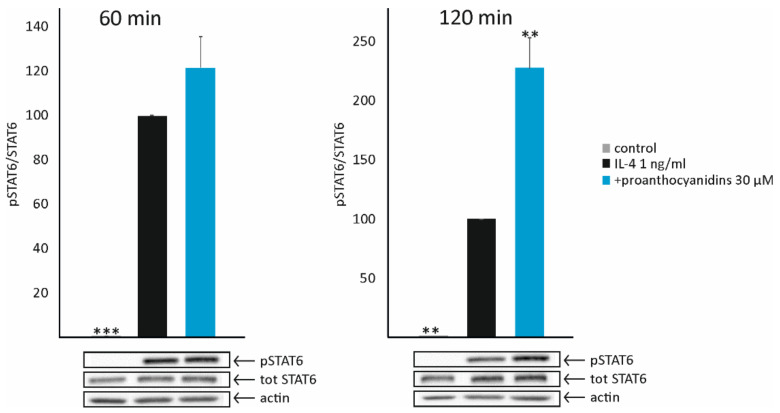
Proanthocyanidins increased the phosphorylation of signal transducer and activator of transcription 6 (STAT6) in J774 macrophages. pSTAT6 protein levels were determined with Western blot and normalized to total STAT6. pSTAT6 expression in IL-4-treated cells was set as 100% (marked as black bar), and the other values are presented in proportion to that value. Actin was used as a loading control. Results are given as the mean + SEM, *n* = 4. One-way ANOVA with Dunnett’s post-test was used in the statistical analysis. Mean values significantly different from the control are marked with ** *p* < 0.01 and *** *p* < 0.001.

To study the effects of resveratrol, kaempferol, and proanthocyanidins in more detail, their effects were measured on two transcription factors important in M2-type activation, namely peroxisome-proliferator-activated receptor gamma (PPARγ) and phosphorylation of signal transducer and activator of transcription 6 (STAT6). Resveratrol and kaempferol significantly enhanced the expression of PPARγ at the 4 h time point, both in unstimulated and in IL-4-stimulated cells (Figure 3). Proanthocyanidins did not have such an effect on PPARγ, but they were found to enhance the phosphorylation of the transcription factor STAT6 in a statistically significant manner (Figure 4).

### 3.3. Resveratrol and Kaempferol Inhibit M1-Type Activation in J774 Macrophages

**Figure 5:**
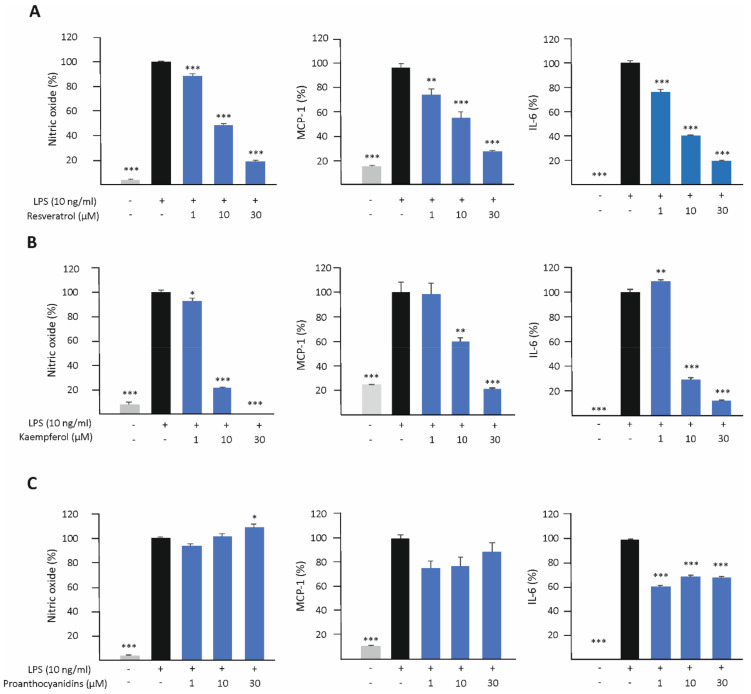
The effects of resveratrol (**A**), kaempferol (**B**) and proanthocyanidins (**C**) on the production of nitric oxide (NO), monocyte chemoattractant protein-1 (MCP-1), and interleukin 6 (IL-6) in J774 macrophages. Cells were cultured with LPS (10 ng/mL) alone or together with resveratrol, kaempferol, or proanthocyanidins for 24 h. The production of NO was measured as the concentrations of nitrite (a stable metabolite of NO) in the culture media by the Griess reaction. MCP-1 and IL-6 concentrations in the culture media were measured by the enzyme-linked immunosorbent assay (ELISA). NO/MCP-1/IL-6 production in LPS-treated cells was set as 100% (marked as black bar), and the other values (untreated control marked as gray bar and the compounds of interest as indicated as blue bars) are presented in proportion to that value. Results are given as the mean + SEM, *n* = 4. One-way ANOVA with Dunnett’s post-test was used in the statistical analysis. Mean values significantly different from LPS-treated cells are marked with * *p* < 0.05, ** *p* < 0.01, and *** *p* < 0.001.

The effects of resveratrol, kaempferol, and proanthocyanidins on M1-type activation was investigated in LPS-stimulated J774 macrophages. The production of nitric oxide (NO), monocyte chemoattractant protein-1 (MCP-1), and interleukin 6 (IL-6) was measured after 24 h of incubation. Resveratrol and kaempferol attenuated the production of NO, MCP-1, and IL-6 in a dose-dependent manner, while the effect of proanthocyanidins was less clear (Figure 5).

### 3.4. Resveratrol and Proanthocyanidins Enhance M2-Type Activation in Human U937 Macrophages

**Figure 6:**
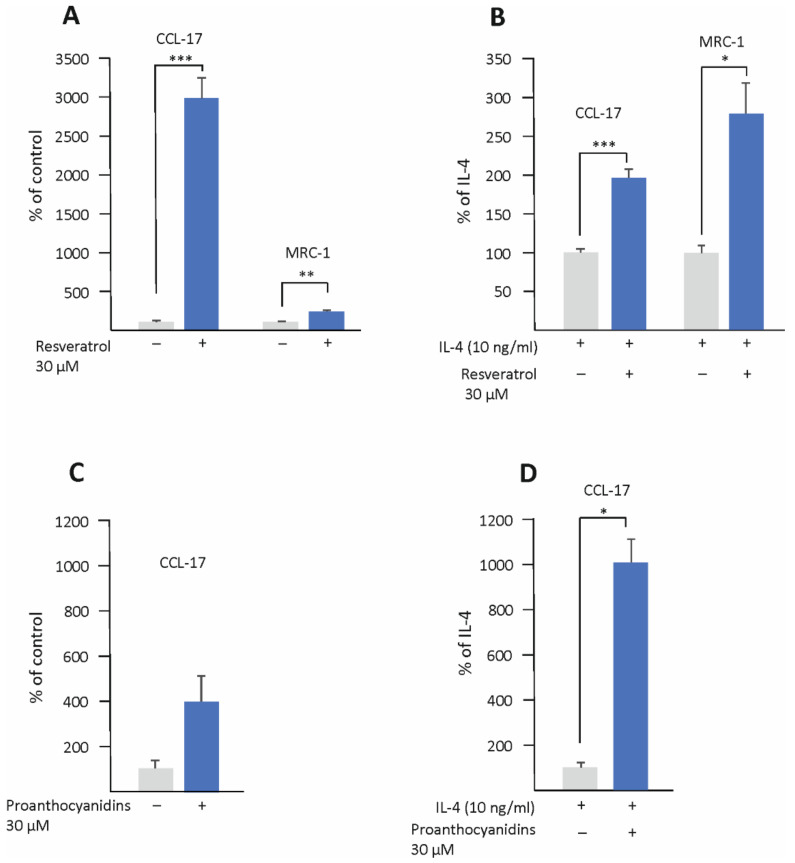
Resveratrol and proanthocyanidins enhanced the expression of CC chemokine ligand 17 (CCL-17) and resveratrol also that of mannose receptor C-type 1 (MRC-1) in human U937 macrophages. Cells were cultured with resveratrol or proanthocyanidins alone (**A**,**C**) or with IL-4 (**B**,**D**) for 24 h. The expressions of CCL-17 and MRC-1 were measured by RT-PCR and normalized against GAPDH. CCL-17/MRC-1 expression in untreated (**A**,**C**) or IL-4-treated (**B**,**D**) cells was set as 100% (marked as gray bar), and the other values (resveratrol or proanthocyanidins, marked as blue bars) are presented in proportion to that value. Results are given as the mean + SEM, *n* = 3–4. Statistical significance of the results was calculated by the *t*-test. Mean values significantly different from the control are marked with * *p* < 0.05, ** *p* < 0.01, and *** *p* < 0.001.

The effect of resveratrol, kaempferol, and proanthocyanidins on M2-type activation was investigated also in human U937 macrophages by measuring their effect on the expression of CC chemokine ligand 17 (CCL-17) and MRC-1. Resveratrol significantly enhanced the expression of CCL-17 and MRC-1 both in unstimulated and in IL-4 -stimulated cells, and proanthocyanidins were found to increase CCL-17 expression in combination with IL-4 (Figure 6). Similar effects were not seen in kaempferol-treated cells.

### 3.5. Resveratrol, Kaempferol, and Proanthocyanidins Inhibit M1-Type Activation in Human U937 Macrophages

**Figure 7:**
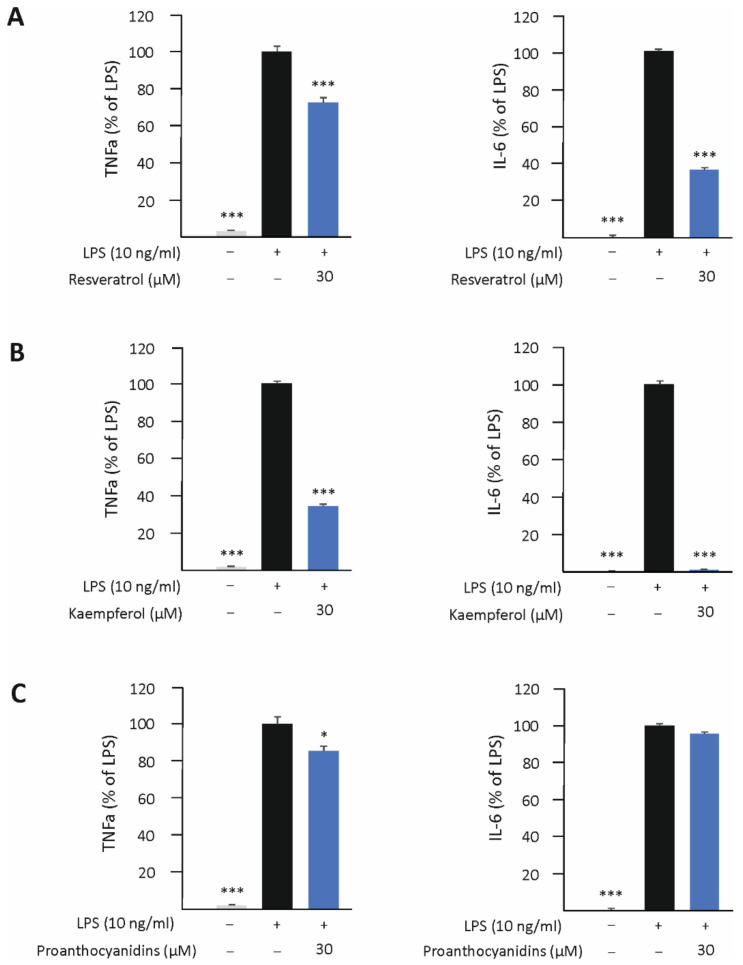
Resveratrol (**A**) and kaempferol (**B**) inhibited the production of tumor necrosis factor alpha (TNF-α) and interleukin 6 (IL-6), and proanthocyanidins (**C**) slightly decreased that of TNF-α in human U937 macrophages. Cells were cultured with resveratrol, kaempferol, or proanthocyanidins and lipopolysaccharide (LPS) for 24 h. Concentrations of TNF-α and IL-6 in the culture media were measured by the enzyme-linked immunosorbent assay (ELISA). TNF-α/IL-6 production in LPS-stimulated cells was set as 100% (marked as black bar), and the other values (resveratrol, kaempferol or proanthocyanidins, marked as blue bars) are presented in proportion to that value. Untreated control is marked as gray bar. Results are given as the mean + SEM, *n* = 4. One-way ANOVA with Dunnett’s post-test was used in the statistical analysis. Mean values significantly different from the control are marked with * *p* < 0.05 and *** *p* < 0.001.

The effect of resveratrol, kaempferol, and proanthocyanidins on M1-type activation in human U937 macrophages was also investigated by measuring their effects on the expression of TNF-α and IL-6 in LPS-stimulated cells. Resveratrol and kaempferol attenuated TNF-α and IL-6 production, while proanthocyanidins had only a slight effect on TNF-α and none on IL-6 production (Figure 7).

## 4. Discussion

Obesity is a global health problem with major health risks. Systemic low-grade inflammation linked to obesity is associated with the development of co-morbidities such as insulin resistance/diabetes, atherosclerosis, hypertension, and some types of cancer [36]. Thus, it is important to find reachable means that could retard the onset and/or progression of low-grade inflammation in overweight and obese people. We and others have shown that lingonberry supplementation has anti-inflammatory properties in models of high-fat diet-induced obesity [22,27,28,37]. To extend the previous lingonberry research data linked to inflammation, we investigated the effect of a group of polyphenol compounds previously identified to be present in lingonberry on macrophage polarization. We found three phenolic compounds, namely resveratrol, kaempferol, and proanthocyanidins, enhance the anti-inflammatory M2-type activation in mouse and human macrophages. Resveratrol and kaempferol were shown to enhance the expression of PPARγ, while proanthocyanidins increased the phosphorylation of STAT6. In addition, resveratrol and kaempferol also effectively inhibited proinflammatory M1-type macrophage activation. Our results extend the previous knowledge of the anti-inflammatory properties of the polyphenols present in lingonberry.

In developing obesity, adipocytes grow, and they suffer from hypoxia and stress [11]. M1-type macrophages are enriched in adipose tissue and contribute to obesity-induced inflammation [15]. Polarization of adipose tissue macrophages towards the inflammatory M1 phenotype is a major factor promoting systemic low-grade inflammation and insulin resistance in obesity [11,15,16]. Obesity-related inflammation appears also in the liver, when the capacity of adipose tissue to store excess energy is reduced along progressing obesity. Excessive fat accumulates in the liver and induces M1-type activation in resident Kupffer cells and infiltrated macrophages, further promoting systemic inflammation and the development of non-alcoholic steatohepatitis [38,39,40]. In the present study, we found three compounds, namely resveratrol, kaempferol, and proanthocyanidins, which are known to be present in lingonberry, to hinder M1-type polarization and, additionally, increase anti-inflammatory M2-type activation of macrophages.

Proanthocyanidins are condensed tannins, more exactly oligo- or polymers of monomeric flavan-3-ols. Most commonly, the constitutive units are (epi)catechins and (epi)gallocatechins [41] (Figure 1). In the present study, proanthocyanidins were found to increase M2-type macrophage polarization evidenced as increased expression of Arg-1 and MRC-1 in murine J774 macrophages and CCL-17 in human U937 macrophages. Proanthocyanidins were discovered to increase the phosphorylation of STAT6, a key transcription factor for M2 polarization. Proanthocyanidins have been reported to have anti-inflammatory properties in vivo and in vitro. For instance, polymeric proanthocyanidin preparation from *Serjania schiedeana* was shown to reduce joint inflammation and increase the levels of the anti-inflammatory type-2 cytokines IL-4 and IL-10 in the joint and spleen in experimentally induced arthritis [42]. Most of the in vitro studies have focused on M1-type macrophage activation [43,44,45,46]. We found only one study in which proanthocyanidins had been investigated on M2-type macrophage activation. In that study, procyanidin B2 was found to enhance the expression of Arg-1, found in inflammatory zone 1 (Fizz1), and chitinase-3-like protein 3 (Ym1) in RAW 264.7 macrophages [47], supporting the current data. In addition, there are two studies in which proanthocyanidin-rich lingonberry extracts have been investigated in LPS-treated murine macrophages and found to suppress M1-type activation [48,49]. Moderate inhibition of M1-type macrophage activation by proanthocyanidins was found also in the present study; however, most importantly, the enhancing effect of proanthocyanidins on M2 activation was discovered, and it is likely to contribute to the anti-inflammatory effects of lingonberry supplementation found previously in obesity models.

Resveratrol (3,5,4′-trihydroxy-*trans*-stilbene; Figure 1) is a phenolic stilbenoid compound and phytoalexin, present commonly in grapes, but found also in lingonberry. Resveratrol is known for its strong antioxidant, antiatherogenic, and antidiabetic properties [50,51,52], and anti-inflammatory effects have also been reported [53]. Resveratrol is considered safe for human use in food supplements [54]. In the present study, we found resveratrol to suppress inflammation by increasing M2-type polarization, as evidenced by increased expression of Arg-1 and MRC-1 and CCL-17 and MRC-1 in murine and human macrophages, respectively. In addition, resveratrol attenuated M1-type activation by decreasing LPS-induced production of NO, MCP-1, and IL-6 in murine J774 macrophages and TNF-α and IL-6 in human U937 macrophages. These results are consistent with previous studies reported in murine and human macrophages [55,56,57]. In a high-fat diet mouse model, resveratrol has been reported to restrict high-fat diet-induced macrophage accumulation in skeletal muscle tissue and switch macrophage polarization from the M1 to the M2 phenotype; accordingly, resveratrol decreased the expression of IL-6, TNF-α, IL-1β, and MCP-1 in skeletal muscle tissue [58]. In human adipose tissue explants, resveratrol treatment reduced the expression of several inflammatory mediators including IL-6, IL-8, MCP-1, IL-1β, and PAI-1 [59]. The results in the current study on the anti-inflammatory effects of resveratrol are consistent, strengthen previous studies, and indicate that resveratrol may contribute to the anti-inflammatory effects of lingonberry supplementation found previously in high-fat diet-induced models of obesity.

Kaempferol (3,4′,5,7-tetrahydroxyflavone; Figure 1) is a natural flavonol, widely present in plants and known to have anti-inflammatory properties. In plants, kaempferol is bound to different sugar molecules and present as glycosides [60,61]. Kaempferol has also been detected in lingonberry [62]. In the present study, kaempferol was found to enhance M2-type macrophage activation evidenced as increased expression of Arg-1 and MRC-1 in J774 cells. Additionally, kaempferol attenuated M1-type activation by decreasing the LPS-induced production of NO, MCP-1, and IL-6 in J774 macrophages and TNF-α and IL-6 in human U937 macrophages. These findings are supported by previous studies, where kaempferol enhanced M2-type polarization in RAW 264.7 mouse macrophages measured as increased expression of Arg-1 and MRC-1 [63]. Kaempferol has also been reported to inhibit LPS-induced M1-type activation in macrophages evidenced as reduced production of M1 markers such as NO and TNF-α [64,65,66,67,68]. In experimental models, kaempferol has been shown to decrease circulating levels of TNF-α and IL-1β in high-cholesterol-fed rabbits [69] and decrease the expression of TNF-α, IL-1β, IL-6, and MCP-1 in the adipose tissue of high-fat diet-fed mice [70]. Results with kaempferol reported in the present study are in accordance with the previous data and indicate that kaempferol present in lingonberries may contribute to the anti-inflammatory effects of lingonberry supplementation found in high-fat diet-induced models of obesity.

To understand the plausible mechanisms behind the detected effects of resveratrol, kaempferol, and proanthocyanidins on M2-type macrophage activation, their effects on two transcription factors important in M2 activation, namely PPARγ and STAT6, were studied. Interestingly, resveratrol and kaempferol were found to increase the expression of PPARγ, while proanthocyanidins increased the phosphorylation of STAT6. There are few studies supporting our results on the increased expression of PPARγ by resveratrol and kaempferol [55,71,72]. To the best of our knowledge, this is the first time when data have been presented to support proanthocyanidins’ promotion of M2 polarization through STAT6 activation. In addition, these different mechanisms of action indicate that, when the three compounds are administered simultaneously, as for instance in lingonberry supplementation, a synergistic effect may be seen.

## 5. Conclusions

The aim of the present study was to investigate the effects of phenolic compounds known to be present in lingonberry on M1/M2 macrophage polarization. We and others have previously shown that lingonberry supplementation prevents low-grade inflammation in experimental models of obesity. Three phenolic compounds previously found to be present in lingonberry, namely resveratrol, kaempferol, and proanthocyanidins, were reported in the present paper to augment anti-inflammatory M2-type macrophage activation. In addition, resveratrol and kaempferol inhibited pro-inflammatory M1-type activation. Resveratrol and kaempferol were shown to enhance the expression of PPARγ, while proanthocyanidins increased STAT6 activation. In further studies, these effects should be confirmed with phenolic compounds purified from lingonberry. In conclusion, the present findings extend the previous knowledge on the anti-inflammatory effects of lingonberry and encourage further studies to support the use of lingonberry and lingonberry-based products as a part of a healthy diet.

## Figures and Tables

**Table 1:** Effects of the twelve phenolic compounds (at 30 µM concentrations) on M2-type activation in murine J774 macrophages.

	Arg-1 (%)	Significance	MRC-1 (%)	Significance
Control	100 ± 5.78		100 ± 5.95	
Resveratrol	161.59 ± 3.78	***	238.8 ± 22.85	***
Piceid	113.48 ± 5.92		79.15 ± 6.17	
Quercetin	120.68 ± 9.83		111.39 ± 7.42	
Kaempferol	174.18 ± 14.21	***	220.67 ± 14.77	***
Proanthocyanidins	340.27 ± 12.30	***	365.50 ± 35.34	***
Delphinidin chloride	92.92 ± 6.24		87.33 ± 6.07	
Cyanidin chloride	91.39 ± 5.08		113.35 ± 8.39	
Benzoic acid	86.62 ± 0.88		98.99 ± 11.67	
Cinnamic acid	117.19 ± 3.05		113.19 ± 2.65	
Coumaric acid	112.93 ± 3.31		119.33 ± 4.48	
Caffeic acid	106.92 ± 7.73		92.5 ± 3.57	
Ferulic acid	100.24 ± 5.34		99.49 ± 8.11	

J774 macrophages were cultured with the compounds of interest for 24 h, and the expressions of arginase-1 (Arg-1) and mannose receptor C-type 1 (MRC-1) were measured by RT-PCR and normalized against GAPDH mRNA. Arg-1/MRC-1 expression in untreated control cells was set as 100%, and the other values are presented in proportion to that value. Results are given as the mean ± SEM, *n* = 4. One-way ANOVA with Dunnett’s post-test was used in the statistical analysis. Mean values significantly different from the control are marked with *** *p* < 0.001.

**Table 2:** The effects of resveratrol, kaempferol, and proanthocyanidins on the expression of Arg-1 and MRC-1 in IL-4-stimulated murine J774 macrophages.

	Arg-1 (%)	Significance	MRC-1 (%)	Significance
IL-4 (1 ng/mL)	100 ± 2.03		100 ± 4.82	
Resveratrol (30 µM)	179.07 ± 4.82	***	204.31 ± 5.14	***
Kaempferol (30 µM)	124.94 ± 3.14	**	134.10 ± 7.00	*p* = 0.22
Proanthocyanidins (30 µM)	230.39 ± 2.96	***	330.27 ± 21.01	***

J774 macrophages were cultured with IL-4 and the compounds of interest for 24 h. Expressions of arginase-1 (Arg-1) and mannose receptor C-type 1 (MRC-1) were measured by RT-PCR and normalized against GAPDH mRNA. Arg-1/MRC-1 expression in IL-4-treated cells was set as 100%, and the other values are presented in proportion to that value. Results are given as the mean ± SEM, *n* = 4. One-way ANOVA with Dunnett’s post-test was used in the statistical analysis. Mean values significantly different from the IL-4 -treated control (IL-4) are marked with ** *p* < 0.01 and *** *p* < 0.001.

## Data Availability

All relevant data are within the paper.
